# Model Clinic to Increase Preventive Screenings Among Patients With Physical Disabilities: Protocol for a Mixed Methods Intervention Pilot Study

**DOI:** 10.2196/50105

**Published:** 2023-10-25

**Authors:** Beatrice Palazzolo, Loretta Carbone, Tyler G James, Robert Heizelman, Ananda Sen, Elham Mahmoudi, Michael McKee

**Affiliations:** 1 Department of Family Medicine, University of Michigan Ann Arbor, MI United States

**Keywords:** clinical decision support, electronic health records, mixed methods, people with physical disabilities, primary care

## Abstract

**Background:**

People with physical disabilities often experience premature multimorbidity and adverse health events. A tailored primary care approach for this vulnerable population that also accounts for social and functional risk factors could promote healthier aging and more equitable health care.

**Objective:**

This project will evaluate the implementation of a health program designed for people with physical disabilities. The proposed evaluation result is to generate the first best-practice protocol focused specifically on developing primary care to help reduce preventable causes of morbidity and improve functioning among people with physical disabilities.

**Methods:**

We will design and implement a pilot health program for people with physical disabilities at a primary care clinic within Michigan Medicine. The health program for people with physical disabilities will be an integrated intervention involving a tailored best practice alert designed to prompt family medicine providers to screen and monitor for common, preventable health conditions. The program will also collect social and functional status information to determine the patient’s need for further care coordination and support. Adult participants from this clinic with identified physical disabilities will be targeted for potential enrollment. To create a quasi-experimental setting, a separate departmental clinic will serve as a control site for comparison purposes. A quantitative analysis to estimate the treatment effect of implementing this health program will be conducted using a difference-in-differences approach. Outcomes of interest will include the use of preventative services (eg, hemoglobin A_1c_ for diabetes screening), social work assistance, and emergency and hospital services. These data will be extracted from electronic health records. Time-invariant covariates, particularly sociodemographic covariates, will be included in the models. A qualitative analysis of patient and health care provider interviews will also be completed to assess the effect of the health program. Patient Health Questionnaire-9 and Generalized Anxiety Disorder 7-item scores will be assessed to both screen for depression and anxiety as well as explore program impacts related to addressing health and functioning needs related to physical disabilities in a primary care setting. These will be summarized through descriptive analyses.

**Results:**

This study was funded in September 2018, data collection started in September 2021, and data collection is expected to be concluded in September 2023.

**Conclusions:**

This study is a mixed methods evaluation of the effectiveness of an integrated health program designed for people with physical disabilities, based on a quasi-experimental comparison between an intervention and a control clinic site. The intervention will be considered successful if it leads to improvements in greater use of screening and monitoring for preventable health conditions, increased social worker referrals to assist with health and functioning needs, and improvements in emergency and hospital-based services. The findings will help inform best practices for people with physical disabilities in a primary care setting.

**International Registered Report Identifier (IRRID):**

DERR1-10.2196/50105

## Introduction

People with disabilities make up approximately 26% (54 million) of the adult population in the United States [1]. Among this population, the most common disabilities are those that impact mobility [1]. Premature multimorbidity and adverse health events are more common among people with physical disabilities compared with the population norm [2-5]. Research shows that healthy aging is strongly influenced by patients’ social, behavioral, and environmental variables [6-8]. However, patients’ information on social and functional status factors is not systematically collected in health care, and therefore, these factors are not considered in medical decision-making aiming to promote healthy aging among this priority population.

There is evidence that primary care generates the largest ability to reduce illness and premature death, as well as providing more equitable health care in populations [9], yet little is known on how this can be achieved effectively for people with physical disabilities [10,11]. The Centers for Disease Control and Prevention (CDC), following the Forman-Hoffman et al [12] report, has provided suggested recommendations to address the existing gaps for individuals with disabilities. These actions include (1) providing a system in which people with physical disabilities at risk for adverse health events, including premature death, can be identified to proactively provide the care and social services that may be needed; (2) tailoring health care services, including health education and prevention, for people with physical disabilities whose needs differ from people without a disability [9]; (3) improving the inclusion of behavioral health interventions for people with physical disabilities; and (4) providing care management for people with physical disabilities by health care staff trained to work with this population [12].

This study aims to explore how care for people with physical disabilities can be fostered and improved through the implementation and comprehensive evaluation of a pilot integrated health program, which will include social support services. To do this, we will (1) design, pilot, and evaluate an accessible, integrated health program for people with physical disabilities at a primary care clinic within Michigan Medicine (ie, Briarwood Family Medicine), scalable for other integrated health centers, and (2) assess how the systematic collection of social and functional status information and use of this information by care managers may reduce adverse health events and improve the social and functional status of people with physical disabilities. Ultimately, our goal is to develop a protocol that provides guidance to other primary care clinics in need of improving the delivery of health care to people with physical disabilities. To our knowledge, this would be the first tailored best practice advisory (BPA) and health care-initiated social intervention designed for people with physical disabilities.

## Methods

### Study Design

#### Overview

**Figure 1:**
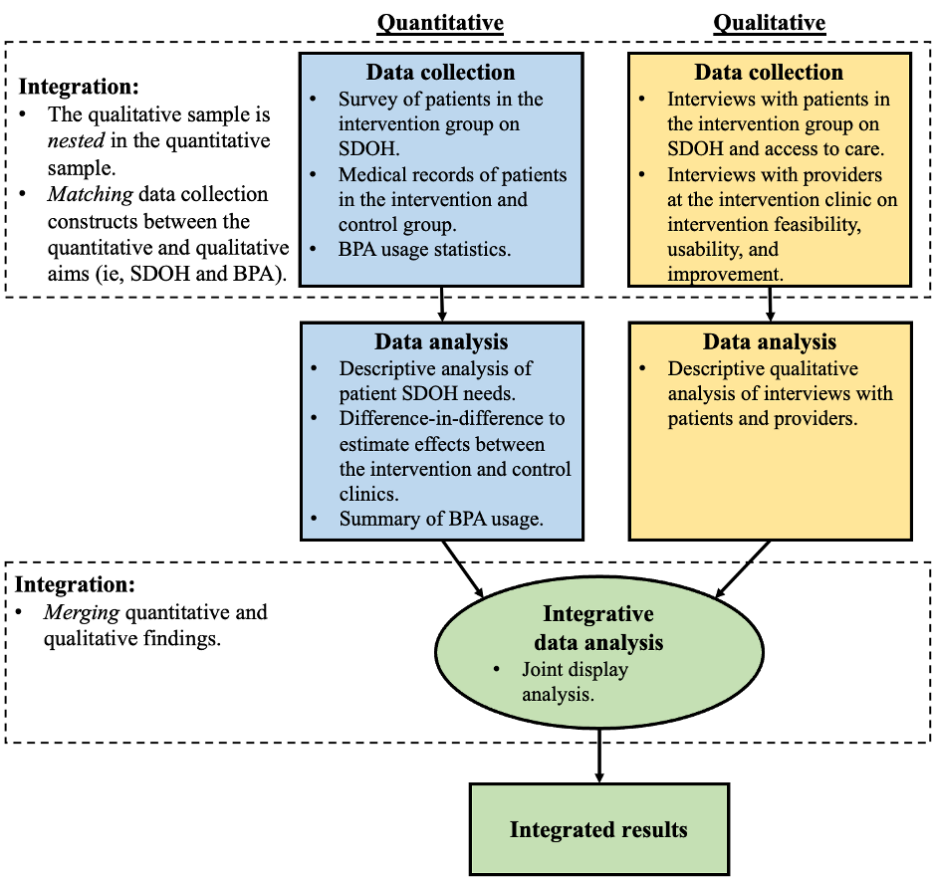
Overview of the pilot intervention study of an integrated care clinic for patients with physical disabilities. BPA: best practice advisory; SDOH: social determinants of health.

This is a pilot intervention study using a convergent mixed methods design. For the quantitative analysis, we will use a quasi-experimental approach, enrolling participants from an intervention site into an integrated health program and using a control site with patients not enrolled in such a program for comparison purposes. Both the intervention and control sites will be separate ambulatory care clinics managed by Michigan Medicine’s Department of Family Medicine. Both sites are located in Southeast Michigan. We will use qualitative methods to better understand social determinants of health among the patient cohort, as well as the perspectives of health care providers on the feasibility and usability of the proposed intervention. An overview of the study design is shown in Figure 1.

#### Intervention

The intervention is based on the paradigm of integrated health programs, which are designed to improve collaboration among primary care providers, social workers, and behavioral health specialists. The co-location of these health professionals at the same clinic allows for higher levels of patient care collaboration; for example, through primary care providers regularly having regular consultations with clinical social workers. This collaboration parallels institution-wide efforts at Michigan Medicine to improve service delivery and integrate medical, social, and behavioral health in a single clinic. This model has been successfully implemented to address significant behavioral health gaps for deaf and hard-of-hearing patients in the Michigan Medicine Family Medicine clinics [13,14].

The intervention has multiple components: (1) development and implementation of an electronic health record (EHR)–based alert to improve preventive screenings among people with physical disabilities; (2) training of providers on how to improve care for people with physical disabilities; and (3) inclusion of a specialized social worker with disability health training.

#### Clinical Decision Support Alert

We will use tailored BPAs in the EHR to provide clinical decision support to primary care providers of people with physical disabilities. This was based on a previously completed study using BPA that increased the rate of hearing screenings and audiology referrals at primary care clinics [15]. BPAs are a type of clinical decision support tool available in Michigan Medicine’s Epic medical record software called MiChart. BPAs are activated in a patient’s medical record when a patient meets eligibility criteria based on factors including diagnostic codes, procedure codes, quality indicators, or laboratory results. BPAs can be used by health care providers to more efficiently order or be reminded about screenings or immunizations. These tools can also aid in documenting medical decision-making. When this BPA is activated, the primary care provider will be prompted to engage the patient to determine the applicability of the BPA by selecting one of the recommended actions or dismissing the alert.

**Figure 2:**
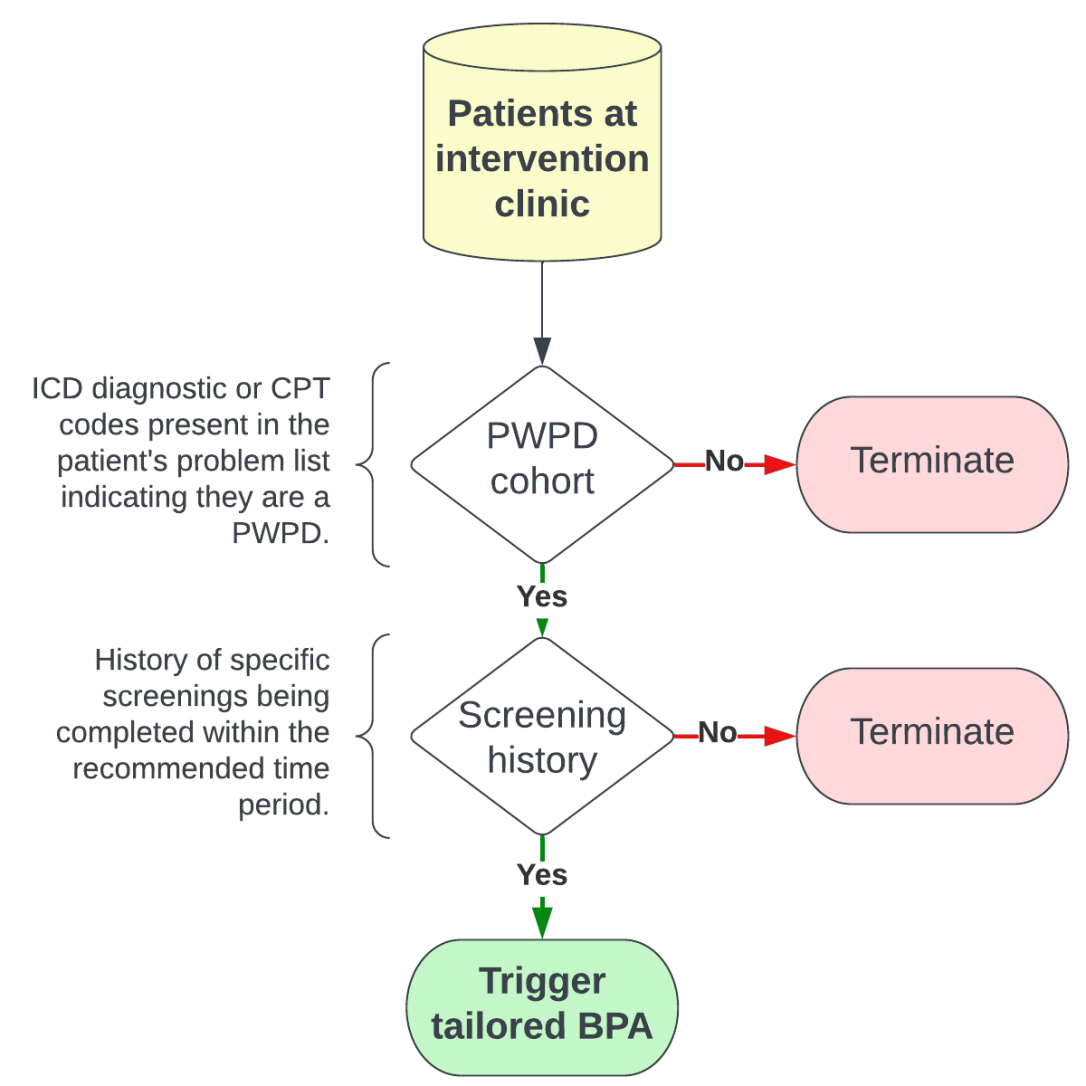
Flowchart of best practice advisory (BPA) activation triggers to improve preventive care for people with physical disabilities (PWPD). CPT: Current Procedural Terminology; ICD: International Classification of Disease.

The BPA for this intervention is designed to prompt preventive and screening services for which there are well-documented disparities for people with physical disabilities [16-18]: hemoglobin A_1c_, bone density DEXA scan, lipid panel, laboratory panels (eg, basic metabolic panel), renal ultrasound, referral to a nutritionist, and referral to a specialized social worker with disability health training. A description of the BPA trigger conditions is available in Figure 2.

**Figure 3:**
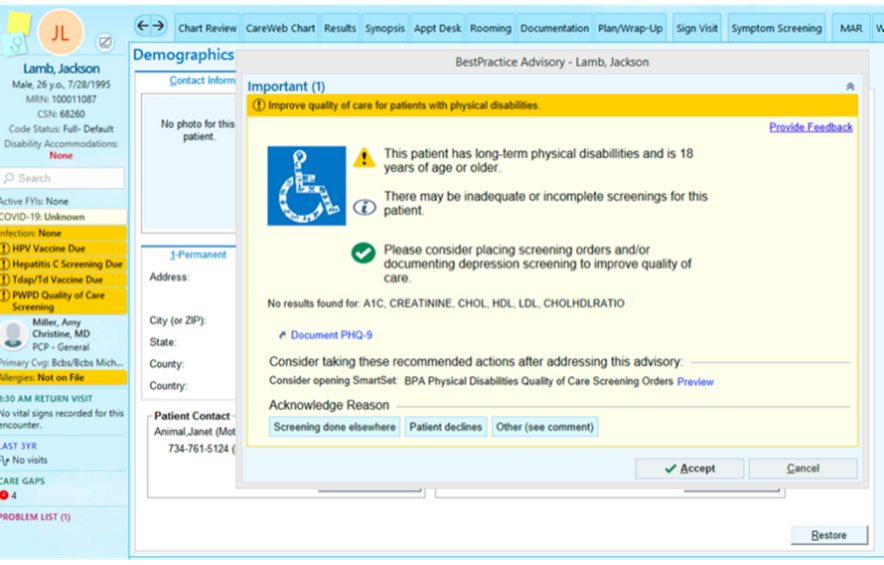
Screenshot of the best practice advisory to improve preventive care for people with physical disabilities (mock patient).

When the BPA is activated for a specific patient, the alert will appear in their medical record in a yellow alert box on the main screen, along with the patient summary (left column; Figure 3). This alert remains active until it is acted on or dismissed by the provider. Available actions are “act on any of the suggested alerts,” “dismiss with an explanation” (eg, patient declines or already completed elsewhere), and “ignore.” Based on the provider’s recommendation and discussion with the patient, the provider can accept the SmartSet proposed by the BPA. The BPA will be pilot-tested by an Epic programming analyst with Michigan Medicine, TGJ, and MM before activation.

#### Provider Training and Communication

Primary care providers at the intervention clinic will be initially trained in intervention design to help reduce preventable causes of morbidity and improve functioning among people with physical disabilities. This training will be done before the implementation of the intervention and will be led by a family medicine clinician-scientist who is an expert in disability health. In addition, throughout the intervention, the research team will meet with the clinic director and other providers to communicate feedback and updates about the intervention.

#### Social Worker

Social work theory posits that an individual’s health may be influenced by organic processes as well as by psychosocial factors in the environment [19]. Therefore, social workers at Michigan Medicine have historically been well integrated with clinics [14], providing case management services, advocacy, care management for maintenance of chronic health conditions, psychotherapy, and additional social support. The social worker for this intervention will work closely with the intervention clinic’s primary care providers to provide additional support in addressing the health, behavioral, functional, and social needs of people with physical disabilities. Patients without any current identified needs will be monitored for any changes with annual BPA prompts.

Patients to be contacted by the social worker will be identified in two ways. Survey participants will complete the seven-item Generalized Anxiety Disorder Scale (GAD-7) and the Patient Health Questionnaire-9 (PHQ-9; Multimedia Appendix 1). The social worker will contact those who indicate moderate to severe anxiety or depression and assess the patient’s need for mental health resources and psychotherapy. A MiChart referral to social work will be created and prepopulated with the following common reasons for referral: caregiver support; durable medical equipment and community referrals; grief and loss; guardianship and advance directives; home modifications; long-term planning; mental health and substance abuse concerns; safety concerns; violence and abuse; and other (with a comment field). Social work interventions will mostly be completed by phone.

#### Patient Cohort

People with physical disabilities will be identified using the International Classification of Disease, Ninth Revision (ICD-9) and International Classification of Disease, Tenth Revision (ICD-10) diagnostic and procedure codes from patients’ problem lists in MiChart (Multimedia Appendix 2). The patient cohort at the intervention clinic will be patients who are identified as people with physical disabilities and who are patients at the intervention clinic (Briarwood Family Medicine). To create a quasi-experimental setting for our intervention, another primary care clinic in the same department will serve as a control clinic (Dexter Family Medicine).

#### Eligibility Criteria

All patients with identified physical disabilities, aged 18 years and older, will trigger the BPA. Using conservative estimates (eg, ICD-9 and ICD-10 codes with strongly associated physical disabilities in Multimedia Appendix 2), we identified 284 patients who were seen in the past 12 months before BPA implementation at the intervention clinic. The characteristics of the patients that were seen in the past 12 months at the intervention clinic were 166 (58.4%) female, 51 (18%) Black, 57 (20.1%) Medicaid insured, and 137 (48.2%) Medicare insured. A similar number of people with physical disabilities is anticipated to be identified in the control clinic.

### Data Sources

#### Overview

This study will use multiple quantitative and qualitative data sources. The alignment of variables and constructs between quantitative and qualitative aims is provided in Textbox 1.

**Quantitative and qualitative data sources and related variables and constructs.**

**Michigan Medicine electronic health record**

- General patient demographics
- Best practice advisory usage

**Patient questionnaire**

- General patient demographics
- Social needs
- Activities of daily living
- Depression and anxiety

**Patient in-depth interviews**

- General facilitators and barriers to accessing primary care
- Patient-provider communication
- Social needs

**Provider in-depth interviews**

- Implementation factors related to using the best practice advisory

#### Michigan Medicine Electric Medical Record (MiChart)

We will download structured clinical data, patient demographics, health care visits, diagnoses, procedures, laboratory orders, and results using the data warehouse at Michigan Medicine. We will also be able to capture the specific use of the BPAs for each patient. Textbox 2 summarizes the important domains that we will use in our analyses. Further, we will use an existing search algorithm [20] in the Electronic Medical Record Search Engine [21], to work with free-text and unstructured clinical documents in electronic medical records in order to identify influential social factors.

**Patient-related domains and variables in structured electronic medical record.**

**Sociodemographic**

- Age
- Race and ethnicity
- Insurance and payor category
- Disadvantage and affluence index (derived from National Neighborhood Data Archive [21])

**Most common comorbidities**

- Driven based on International Classification of Diseases (Ninth Revision before October 2015 or Tenth Revision after October 2015) coding algorithms for Elixhauser comorbidities (29 conditions) from inpatient, outpatient, or emergency visits during the previous 12 months

**Medication count**

- 1-5
- 6-10
- 11-20
- >20

**Use of agents**

- Antithrombics
- Chemotherapeutics
- Hypoglycemics
- Insulins
- Narcotics
- Opioids, etc

**Abnormal biomarkers**

- High-density lipoprotein
- Hemoglobin A_1c_
- Lactate dehydrogenase, etc

#### Patient Questionnaire

A short questionnaire (“Partners in Health”) will be prospectively collected from people with physical disabilities who meet our inclusion criteria at the time of the BPA implementation. The collected data are related to basic social needs (eg, ability to pay and social and family support; Multimedia Appendix 1).

#### Patient In-Depth Interviews

To understand barriers and facilitators to accessing primary health care among people with physical disabilities, we will recruit people with physical disabilities who complete the aforementioned patient questionnaire and consent to being recontacted for a one-hour interview. The interview will be semistructured, guided by an interview guide (Multimedia Appendix 3), and implemented by the intervention social worker (LC) or mixed methods methodologist (TGJ).

#### Provider In-Depth Interviews

Provider interviews will be conducted to understand usability, feasibility, and implementation constructs related to the use of the BPA alert. The interview guide (Multimedia Appendix 4) is informed by the Consolidated Framework of Implementation Research (CFIR) [22]. These interviews will be conducted by MM, a family medicine clinician-scientist, to aid in establishing rapport and trust.

### Quantitative Analysis

#### Preliminary Analyses

Before being analyzed, the data will be explored to identify any missingness, which will be handled appropriately (eg, using imputation techniques or keeping complete cases only), based on its extent, impact on the sample size, and missing data pattern.

The characteristics of patients in the intervention group will be summarized using descriptive statistics. We will compare the characteristics of the intervention clinic with those of the control clinic and test for relevant associations using Student's *t* test or Wilcoxon signed-rank test for continuous variables and chi-square or Fisher exact tests for categorical variables as appropriate.

If this preliminary exploration of the full sample shows a lack of balance between the groups, we will apply inverse propensity weighting to calculate a weight defined as the inverse probability of observing the given sample [23-25].

To verify the comparability of the intervention and the control sites, which is a key requirement for our quantitative intervention effectiveness analysis, we will also analyze data on the main outcomes of interest dating back to three years before the launch of the intervention.

In addition, preliminary analyses will also include summarizing the patient questionnaire data, mental health scores (GAD-7 and PHQ-9 overall scores), and BPA usage data. The questionnaire data will be described and may be merged with EHRs to be further used in qualitative analyses. The BPA usage data analysis will be used to investigate the (1) provider type acting on the BPA, (2) type of appointment when the BPA is firing, and (3) what actions were taken with respect to the BPA order.

#### Difference-in-Differences (DD) Intervention Effectiveness Analysis

The primary health–based outcomes assessed will be used for preventative services (eg, diabetes and lipid screening rates and hemoglobin A_1c_) and the number of emergency room or hospital visits. The primary behavioral outcomes will be based on the changes in the PHQ-9 and GAD-7 overall scores before and after the intervention (after one month).

In order to evaluate the effect of the integrated health program on these outcomes, we model these outcomes using a DD approach [26]. DD is a statistical technique applied to longitudinal data that attempts to find a causal effect between an intervention and a control group in a quasi-experimental setting before and after a certain intervention is implemented. It assumes that, if the groups were similar based on observable characteristics and preintervention trends, any differences between them with respect to the outcomes of interest can be attributed to the intervention itself, rather than to systematic, preexisting differences. The average change in the outcome in each group is compared to estimate an average intervention effect for the intervention group (known as the average treatment effect for the treated).

**Figure 4:**
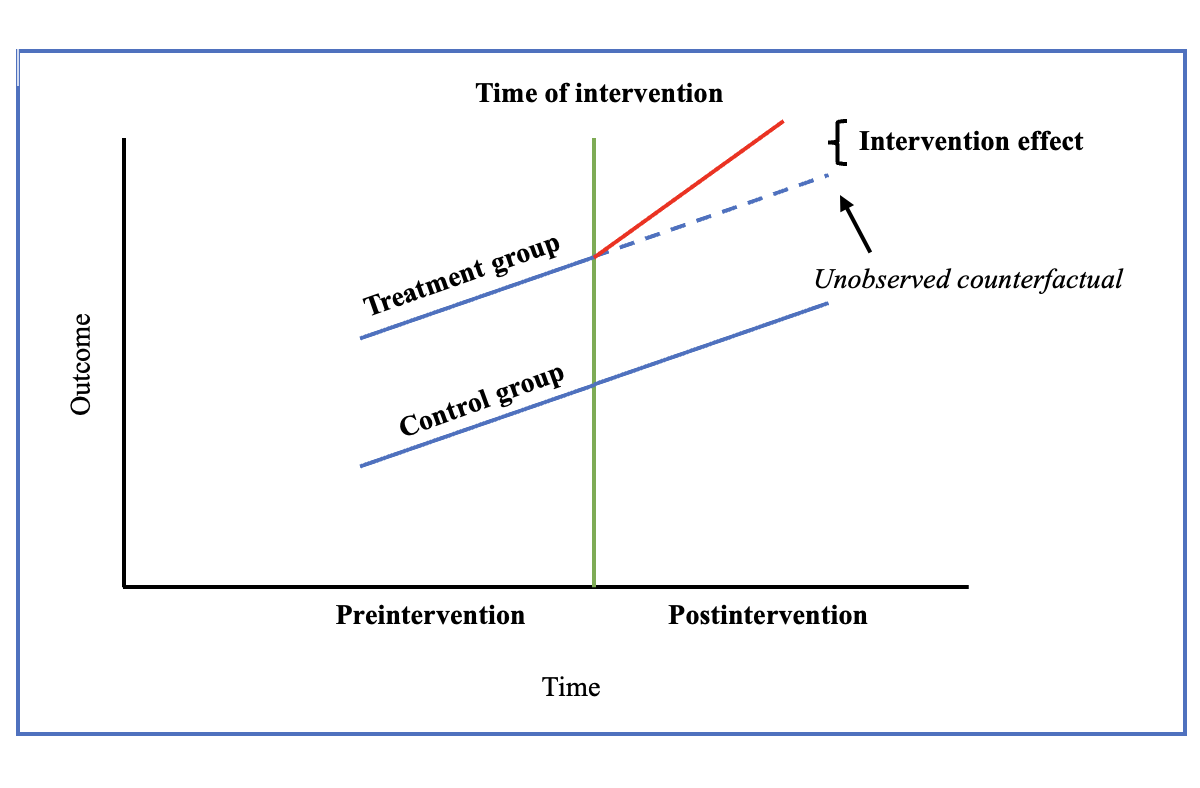
Graphical representation of difference-in-differences approach’s intervention effect and parallel trend assumption.

The key assumption DD relies on is, thus, the presence of parallel trends; in the absence of the intervention, the treatment and control groups would continue to follow analogous trends with respect to the outcomes of interest. The postintervention, parallel trend in the intervention group would be an unobserved counterfactual path. Additionally, in the context of DD, particular attention to meeting the stable unit treatment value assumption is also required; the composition of treatment and control groups should remain stable over time to avoid spillover effects [9]. Figure 4 provides a simple graphical representation of the intervention effect and the parallel trend assumption.

In the simple case of 2 time periods (1 pre- and 1 postintervention period), DD involves building a regression model with the following key predictors: a binary time covariate (before and after the intervention), a binary intervention covariate (treatment and control group), an interaction term between the 2 variables (ie, the DD estimator), and any time-varying covariates that may affect the outcome’s trend over the observed time period without being themselves affected by the intervention. Table S1 in Multimedia Appendix 5 outlines the list of outcomes and related models.

All statistical analyses will be performed using the software RStudio [27].

#### Qualitative Data Analysis

Qualitative data will be collected from the semistructured interviews with people with physical disabilities and primary care providers at the intervention clinic. Interviews will be audio-recorded to facilitate transcription by a Health Insurance Portability and Accountability Act (HIPAA)–compliant transcription service. Transcriptions will be imported into MAXQDA 2022 (Verbi Software) to conduct qualitative analysis.

The research team will code patient and provider interviews separately, as the codes that are developed will be different; provider interview questions are based on an implementation framework, while the patient interview questions are focused on quality of life and clinic access. The overarching qualitative strategy that will be used in this study is the qualitative description, as described by Sandelowski [28,29]. In qualitative description, the researchers seek to keep the analysis closely grounded in the participants’ words while also using theory and conceptual models as appropriate.

A preliminary codebook will be developed through multiple members of the research team open-coding the same transcript from each group and meeting to discuss coding decisions. This process encourages a collaborative process of developing codes and code definitions across multiple coders who will independently code the transcripts. In addition, transcripts from the provider group will be coded using codes aligned with the CFIR [22]. Additional codes may be added to the codebook as the analysis progresses; in this case, the qualitative analysis team will meet to discuss the appropriateness of developing a new code, the name of the code, and its definition. Previous transcripts will be rereviewed to ensure the same codebook is applied to each transcript. Throughout the analysis, coders will be encouraged to memo transcripts with overarching thoughts, theme development, and other analytic insights [30].

Themes will be developed after the coding is complete. The research team will review the developed memos, code segments, and code relations, in addition to “focusing strategies” as described by Saldana [30]. For example, we will use the “touch test” to ensure that the developed themes are conceptually relevant and not just a description of a code.

### Ethical Considerations

This study was approved by the University of Michigan institutional review board (HUM00189835). The project was given an exemption from the institutional review board because the information obtained from the survey is recorded in such a manner that the identity of the survey participants cannot readily be ascertained. The consent form included a statement that deidentified participant information may be shared in publications or for research purposes. There was a hybrid informed consent process. Paper consent forms and surveys were sent to all potential participants, which could be returned through an included envelope. Participants were also given the option to complete an electronic consent form and survey through Research Electronic Data Capture (REDCap). An incentive of US $2 cash was included with the mailed paper survey. Interview participants were given a gift card worth US $10 for their participation.

## Results

The BPA was activated in the intervention clinic’s EHR on September 16, 2021. The research team has had monthly meetings with the clinical director and the Department of Family Medicine’s Director of Medical Informatics since implementation. The intervention patient cohort was sent the survey on May 5, 2022, and data collection for this source was completed on November 1, 2022.

Questionnaire data, including anxiety and depression overall scores (ie, GAD-7 and PHQ-9), and basic needs access (ie, “Partners in Health”; Multimedia Appendix 1), as well as BPA usage data, will be explored through descriptive statistics in the fall of 2023. Preliminary analyses to test assumptions about the comparability of the intervention and the control clinics will be conducted in late 2023 using data from the EHR, provided by the Michigan Medicine Data Office for Clinical and Translational Research. Interviews with people with physical disabilities were completed in December 2022, and formal data analysis will begin in the fall of 2023. During the spring and summer of 2023, we will meet with health care providers at the intervention clinic to conduct the semistructured interviews.

The results from our quantitative and qualitative analyses will be combined using mixed methods integrative data analysis [31,32]. These integrated results will be used to advance knowledge of best practices in the area of primary care and well-being for people with physical disabilities and pave the way for a more comprehensive and equitable approach to care for people with physical disabilities. Our findings will be submitted for publication in peer-reviewed journals once the study is completed.

## Discussion

### Overview

The quantitative results from the primary aim of this study, which is to measure the effectiveness of a pilot model clinic designed for people with physical disabilities, will advance knowledge on best practices for caring for a population that is disenfranchised in health care. Supplementing the primary findings with the aid of qualitative methods, our secondary focus on social support will allow us to explore the full extent of the role that an integrative approach can have in improving the health and well-being of people with physical disabilities. The integration of different data points will help determine if the intervention can be successfully implemented into a primary care setting. Second, it will help inform whether the intervention leads to (1) an improved use of tailored screening and monitoring needed for this population; (2) an increased social worker referral to assist with health and functioning needs related to their physical disabilities; and (3) reduced preventable causes of morbidity and improved functioning among people with physical disabilities. Thus, the merit of a combined quantitative and qualitative approach in the particular context of researching and improving care for people with physical disabilities is that together these methodologies will allow us to explore both the measurable, physical impact of an integrated approach to care for people with physical disabilities and the less measurable, nuanced social, behavioral, and environmental factors, which are better captured in questionnaires and interview settings than chart abstraction.

The mixed methods findings of this study will be used to improve the proposed clinical model and identify further opportunities to develop a comprehensive protocol for primary care clinics to improve the care of people with physical disabilities. In line with previous literature observing the key impact of primary care and of people with physical disabilities’ social, behavioral, and environmental factors on the health of people with physical disabilities [9], our uniquely integrated intervention has the potential to significantly advance care and preventive practices for people with physical disabilities. Integrated findings will also be used to improve the BPA. This protocol will also provide a framework for the actions recommended by the CDC to address the existing health care gaps for individuals with disabilities. The overarching clinical model, with recommended improvements, may be tested in future clinical trials.

Some of the major strengths that we anticipate for this study are the presence of a similar number of eligible patients with physical disabilities in the intervention and control sites, the sites being part of the same health group and based in the same geographic area, which facilitates comparisons and monitoring to prevent spillover effects, and the presence of all health professionals involved in the integrated health program within each site, which enables close collaboration during the implementation period.

Despite the considerable benefit of the knowledge gained from this study and the noted strengths, this study presents some possible limitations. DD, the main approach adopted to test intervention effectiveness, will allow us to estimate a causal effect only if its assumptions are satisfied. The assumptions that could represent the biggest threat to validity in our case are low between-group similarity, parallel trends preintervention, and stable group composition pre-post intervention (including, ideally, during the follow-up period). If required, we will attempt to address a lack of balance with respect to the covariates of interest through inverse propensity weighting and any potential failure to meet other DD assumptions by adopting alternative modeling methodologies.

In addition, the sample size in the qualitative aim is a potential risk. Adequate sample size, prolonged engagement, and participant trust are pivotal to ensuring the credibility of results [33]. To help address these factors, medical providers will be interviewed by a medical provider (MM), while interviews with patients will be conducted by a trained clinical social worker (LC) and a community-engaged, mixed methods research methodologist (TGJ).

Finally, because this is a pilot study, we are limiting its implementation to one intervention and one control site. While having more sites would provide us with a larger sample size and a stronger claim for external validity, trialing the intervention on a smaller, local scale will allow us to follow the program closely and develop it optimally, with the aim of expanding its scope in the future and working on a more extensive analysis.

Overall, this study will integrate findings from multiple data sources to best assess the feasibility and potential effectiveness of the proposed model clinic for primary care tailored to people with physical disabilities. Our findings will inform health care services for people with physical disabilities and lead to a more equitable paradigm with respect to primary care for people with physical disabilities.

### Conclusions

Currently, there are no formal guidelines to ensure that people with physical disabilities, a particularly vulnerable population, receive appropriate, tailored care for their needs and susceptibility to premature multimorbidity and adverse health events. This is aggravated by the lack of available data on the social and functional context (known factors that contribute to healthy aging) of people with physical disabilities. Research that links all these aspects and tests effective solutions that can be feasibly scaled is crucial to changing this and establishing best practices. The results and protocol generated from this quasi-experimental study aim at achieving this in a robust manner, providing a contribution to the literature as well as making an impact on the national health system.

## Appendix

Multimedia Appendix 1 Patient Questionnaire.

Multimedia Appendix 2 Diagnostic and Procedure Codes to Identify Patient Cohort.

Multimedia Appendix 3 Patients with Physical Disabilities Interview Guide.

Multimedia Appendix 4 Provider Interview Questions.

Multimedia Appendix 5 Outcomes and models for the intervention effectiveness analysis.

## Abbreviations

BPA: best practice advisory
CDC: Centers for Disease Control and Prevention
CFIR: Consolidated Framework for Implementation Research
DD: difference-in-differences
EHR: electronic health record
GAD-7: 7-item Generalized Anxiety Disorder Scale
HIPAA: Health Insurance Portability and Accountability Act
ICD-10: International Classification of Disease, Tenth Revision
ICD-9: International Classification of Disease, Ninth Revision
PHQ-9: Patient Health Questionnaire-9
REDCap: Research Electronic Data Capture

## Data Availability

The data sets currently being generated or analyzed during this study will first be curated and anonymized through the assistance of the data archive staff at the University of Michigan’s Inter-university Consortium for Political and Social Research. Once this is completed, this will be made publicly available.
