# Supplementary material for: Model Clinic to Increase Preventive Screenings Among Patients With Physical Disabilities: Protocol for a Mixed Methods Intervention Pilot Study
Source: JMIR Res Protoc. 2023 Oct 25;12:e50105. doi: 10.2196/50105 (PMC10632921; doi:10.2196/50105)
Supplement: Multimedia Appendix 1 [file resprot_v12i1e50105_app1.docx]

**Appendix 1: Patient Questionnaire**

**PARTNERS IN HEALTH QUESTIONNAIRE**

At Michigan Medicine, we believe that basic needs like access to healthy foods and a safe, warm home influence your overall health. We would like to ask you about your basic needs, so that our social worker can connect you to resources if wanted or needed.

Please check your answers to the statements and questions below. If you are not the patient, please answer questions 1-14 as if you were the patient.

1. Food: Within the last 12 months, you worried that your food would run out before you got money to buy more.

☐ Never true ☐ Sometimes true ☐ Often true

1. Food: Within the past 12 months, the food you bought just didn’t last and you didn’t have money to get more.

☐ Never true ☐ Sometimes true ☐ Often true

1. Transportation: In the last 12 months, has lack of transportation kept you from medical appointments or from getting medications?

☐ Yes ☐ No

1. Transportation: In the past 12 months, has lack of transportation kept you from meetings, work or getting things needed for daily living?

☐ Yes ☐ No

1. Healthcare financial strain: In the past 12 months, did you NOT see a doctor when you needed to or skip medications to save money?

☐ Yes ☐ No

1. Utilities: In the last 12 months, has the utility company shut off your service for not paying your bills?

☐ Yes ☐ No

1. Housing: In the next 2 months, are you worried that you may not have stable housing?

☐ Yes ☐ No

1. Career: Do you need help finding a local career center and/or job training?

☐ Yes ☐ No

1. Elder/Child care: In the last 4 weeks, did getting elder care or child care make it difficult to work or study?

☐ Yes ☐ No

1. Social: How often do you feel isolated from others?

Relationships: Within the last year, have you been afraid of someone close to you?

☐ Never ☐ Rarely ☐ Sometimes ☐ Often ☐ Always

1. Relationships: Within the last year, have you been humiliated or emotionally abused in other ways by someone close to you?

☐ Yes ☐ No

1. Relationships: Within the last year, have you been kicked, hit, slapped or otherwise physically hurt by someone close to you?

☐ Yes ☐ No

1. Relationships: Within the last year, have you been raped or forced to have any kind of sexual activity by someone close to you?

☐ Yes ☐ No

1. Your relationship to the patient:

☐ Patient/Self ☐ Parent/Step-parent ☐ Legal Guardian

☐ DPOA for healthcare ☐ Other:

**FUNCTIONAL STATUS QUESTIONNAIRE**

This questionnaire may be completed over the phone by calling 734-232-0291.

Please complete the following:

| **Which of the following statements describes your work situation during the past month?** | |
| --- | --- |
|  | 1. Working full-time |
|  | 1. Working part-time |
|  | 1. Unemployed, looking for work |
|  | 1. Unemployed because of my health |
|  | 1. Retired because of my health |
|  | 1. Retired for some other reason |

| During the past month, how many days did you cut down on the things you usually do for one-half day or more because of your illness or injury? | | | | | | | | | | | | | | | | | | | | | | | | | | | | | | | |
| --- | --- | --- | --- | --- | --- | --- | --- | --- | --- | --- | --- | --- | --- | --- | --- | --- | --- | --- | --- | --- | --- | --- | --- | --- | --- | --- | --- | --- | --- | --- | --- |
| 0 | 1 | 2 | 3 | 4 | 5 | 6 | 7 | 8 | 9 | 10 | 11 | 12 | 13 | 14 | 15 | 16 | 17 | 18 | 19 | 20 | 21 | 22 | 23 | 24 | 25 | 26 | 27 | 28 | 29 | 30 | 31 |

| **During the past month have you:** | Usually did with no difficulty | Some difficulty | Much difficulty | Usually did not do because of health | Usually did not do for other reasons |
| --- | --- | --- | --- | --- | --- |
| 1. Had difficulty visiting with friends or relatives? |  |  |  |  |  |
| 1. Had difficulty participating in community activities, such as religious services, social activities, or volunteer work? |  |  |  |  |  |
| 1. Had difficulty taking care of other people such as family members? |  |  |  |  |  |

| **During the past month have you:** | All of the time | Most of the time | Some of the time | None of the time |
| --- | --- | --- | --- | --- |
| 1. Isolated yourself from people around you? |  |  |  |  |
| 1. Acted affectionately toward others? |  |  |  |  |
| 1. Acted irritable toward those around you? |  |  |  |  |
| 1. Made unreasonable demands on your family and friends? |  |  |  |  |
| 1. Gotten along well with other people? |  |  |  |  |

| **During the past month, how satisfied were you with your sexual relationships?** | |
| --- | --- |
|  | 1. Very satisfied |
|  | 1. Satisfied |
|  | 1. Not sure |
|  | 1. Dissatisfied |
|  | 1. Very dissatisfied |
|  | 1. Did not have any sexual relationships |

| **During the past month, how often did you get together with friends or relatives, such as going out together, visiting each other’s homes, or talking on the telephone?** | |
| --- | --- |
|  | 1. Every day |
|  | 1. Several times a week |
|  | 1. About once a week |
|  | 1. 2-3 times a month |
|  | 1. About once a month |
|  | 1. Not at all |

| **How do you feel about your health?** | |
| --- | --- |
|  | 1. Very satisfied |
|  | 1. Satisfied |
|  | 1. Not sure |
|  | 1. Dissatisfied |
|  | 1. Very dissatisfied |

**PLEASE COMPLETE THE FOLLOWING SECTIONS IF YOU DO NOT USE A WHEELCHAIR TO GET AROUND**

Physical Function/Activities of Daily Living

| **During the past month have you had difficulty with:** | Usually did with no difficulty | Some difficulty | Much difficulty | Usually did not do because of health | Usually did not do for other reasons |
| --- | --- | --- | --- | --- | --- |
| 1. Taking care of yourself, that is, eating, dressing or bathing? |  |  |  |  |  |
| 1. Moving in or out of a bed or chair? |  |  |  |  |  |
| 1. Walking indoors, such as around your home? |  |  |  |  |  |
| 1. Walking several blocks? |  |  |  |  |  |
| 1. Walking one block of climbing one flight of stairs? |  |  |  |  |  |
| 1. Doing work around the house, such as cleaning, light yard work or home maintenance? |  |  |  |  |  |
| 1. Doing errands such as grocery shopping? |  |  |  |  |  |
| 1. Driving a car or using public transportation? |  |  |  |  |  |
| 1. Doing vigorous activities such as running, lifting heavy objects or participating in strenuous sports? |  |  |  |  |  |

| **If you were employed during the past month, how was your work performance?** | All of the time | Most of the time | Some of the time | None of the time |
| --- | --- | --- | --- | --- |
| 1. Done as much work as others in similar jobs? |  |  |  |  |
| 1. Worked for short periods of time or taken frequent rests because of your health? |  |  |  |  |
| 1. Worked a regular number of hours? |  |  |  |  |
| 1. Done your job as carefully and accurately as others with similar jobs? |  |  |  |  |
| 1. Worked at your usual job, but with some changes due to your health? |  |  |  |  |
| 1. Feared losing your job because of your health? |  |  |  |  |

This questionnaire may be completed over the phone by calling 734-232-0291.


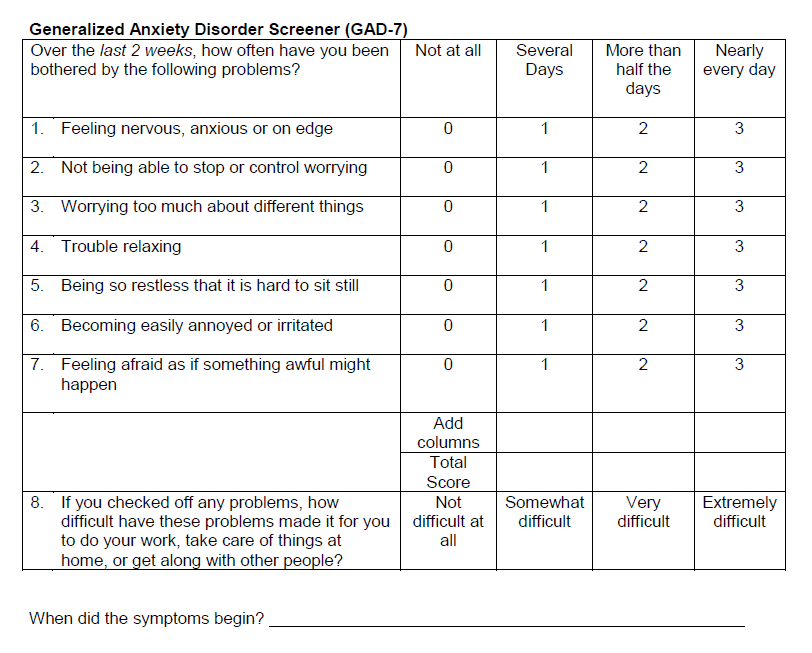


**Patient Health Questionnaire**

Over the last 2 week, how often have you been bothered by any of the following problems? (Circle the number that best describes your answer).

|  | | **Not at all** | **Several Days** | **More than half the days** | **Nearly every day** |
| --- | --- | --- | --- | --- | --- |
| 1. Little interest or pleasure in doing things. | | 0 | 1 | 2 | 3 |
| 1. Feeling down, depressed, or hopeless. | | 0 | 1 | 2 | 3 |
| 1. Trouble falling or staying asleep or sleeping too much. | | 0 | 1 | 2 | 3 |
| 1. Feeling tired or having little energy. | | 0 | 1 | 2 | 3 |
| 1. Poor appetite or overeating. | | 0 | 1 | 2 | 3 |
| 1. Feeling bad about yourself – or that you are a failure or have let yourself or your family down. | | 0 | 1 | 2 | 3 |
| 1. Trouble concentrating on things, such as reading the newspaper or watching television. | | 0 | 1 | 2 | 3 |
| 1. Moving or speaking so slowing that other people could have noticed. Or the opposite – being so fidgety r restless that you have been moving around a lot more than usual. | | 0 | 1 | 2 | 3 |
| 1. Thoughts that you would be better off dead, or of hurting yourself. | | 0 | 1 | 2 | 3 |
|  | Add columns | |  |  |  |
|  | | TOTAL: |  |  |  |
| 1. If you checked off any problems, how difficult have these problems made it for you to do your work, take care of things at home, or get along with other people? | |  | Not at all difficult  Somewhat difficult  Very difficult  Extremely difficult | |  |
|  |  |  |  |  |  |
|  |  |  |  |  |  |
|  |  |  |  |  |  |
| Copyright 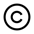1999 Pfizer Inc. All rights reserved. Reproduced with permission. PRIME-MD 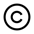is a trademark of Pfizer, Inc. A2663B 10-04-2005 | | | | | |
