# Supplementary material for: Model Clinic to Increase Preventive Screenings Among Patients With Physical Disabilities: Protocol for a Mixed Methods Intervention Pilot Study
Source: JMIR Res Protoc. 2023 Oct 25;12:e50105. doi: 10.2196/50105 (PMC10632921; doi:10.2196/50105)
Supplement: Multimedia Appendix 2 [file resprot_v12i1e50105_app2.docx]

**Appendix 2: Diagnostic and Procedure Codes to Identify Patient Cohort**

**ICD-9/ICD-10 Diagnostic Codes:**

| 340 | G82.51 | G37.2 | R53.2 |
| --- | --- | --- | --- |
| 341 | G82.52 | G37.3 | Y81.2 |
| 341.1 | G82.53 | G37.4 | Z44.1 |
| 341.2 | G82.54 | G37.5 | Z44.9 |
| 341.20 | G83.3 | G37.8 | Z47.81 |
| 341.21 | G83.8 | G37.9 | Z47.89 |
| 341.22 | G83.9 | G71.0 | Z73.6 |
| 341.8 | Q05.4 | G72.3 | Z74.0 |
| 341.9 | Q05.6 | G80.0 | Z74.09 |
| 359 | Q05.7 | G80.1 | Z74.1 |
| G12 | Q05.8 | G80.2 | Z89.0 |
| G35 | Q05.9 | G80.3 | Z89.1 |
| G36 | Q07.02 | G80.9 | Z89.2 |
| G36.0 | Q07.03 | G81.9 | Z89.4 |
| G36.1 | Q76.0 | G82 | Z89.5 |
| G36.8 | Q77.5 | G82.2 | Z89.6 |
| G36.9 | Q78.8 | G82.20 | Z89.9 |
| G37 | Q80.8 | G82.5 | Z97.1 |
| G37.0 | R26.9 | G82.50 | Z99.3 |
| G37.1 | R29.5 |  |  |

**Procedure Codes:**

| 0Y6J0Z3 |
| --- |
| 84.14 |
| 84.1 |
