# Supplementary material for: Model Clinic to Increase Preventive Screenings Among Patients With Physical Disabilities: Protocol for a Mixed Methods Intervention Pilot Study
Source: JMIR Res Protoc. 2023 Oct 25;12:e50105. doi: 10.2196/50105 (PMC10632921; doi:10.2196/50105)
Supplement: Multimedia Appendix 3 [file resprot_v12i1e50105_app3.docx]

**Appendix 3: Patients with Physical Disabilities Interview Guide**

**First, we would like some general information about you:**

Do you identify as a person with a disability?

What is/are the major health condition(s) that affect you?

PROBE: Is that all?

PROBE: At what age were you diagnosed with your physical disability or X diagnosis?

How has your health changed over the time since the diagnosis?

PROBE: Any other changes that you can think of?

Do you have a regular caregiver?

IF YES, is your caregiver a formal paid caregiver, informal or both?

IF YES, what tasks does the caregiver(s) help with?

What equipment do you use because of your physical disability or X diagnosis?

Are you currently employed?

IF YES: Can you tell me a little about your work?

PROBE: Is it full-time or part-time?

PROBE: Do you work from home or go to a workplace?

IF NO: Are you retired or on disability or can you explain why you are not working?

**We also want to hear about your experience in the health care system. Please think of your last appointment at [clinic name] Family Medicine with a doctor, physician’s assistant or nurse practitioner. Who was your last appointment with?**

- Doctor
- Physician Assistant
- Nurse Practitioner

On a scale of 1 to 5, where 1 is poor and 5 is excellent, how would you rate your communication with the doctor/physician assistant/nurse practitioner?

| - 1. Poor | - 2. Not good | - 3. Unsure | - 4. Very good | - 5. Excellent |
| --- | --- | --- | --- | --- |

How did you decide this score? What went well? What went poorly?

What did you like about the doctor/physician assistant/nurse practitioner?

Have you experienced any difficulties with scheduling to see your primary care doctor at [clinic name] Family Medicine?

How has this impacted your care?

For interviewees who have experienced the BPA: I see that during a visit with your provider, your provider ordered XXX (e.g., referral to social work, DEXA scan, etc.). Can you tell me more about that visit?

PROBE: Provider communication/education with patient

PROBE: Patient concerns/questions/involvement

If referred to social work: What has your experience been like working with the social worker?

PROBE: Has it impacted the level of resources they have related to social needs?

PROBE: Patient-social worker working alliance.

WE ARE INTERESTED IN PHYSICAL ACCESS TO THE CLINIC:

How do you normally get to the clinic/your medical appointments?

Did you feel that your last health care visit with your primary care provider was accessible?

IF NOT: what did you feel was needed?

IF YES: what did you feel helped with access?

WE ARE ALSO INTERESTED IN WHETHER YOU FEEL RESPECTED BY YOUR PRIMARY CARE PROVIDER:

Did you feel that you were treated with respect at your last health care visit with your primary care provider?

IF NOT: what did you feel was not respectful?

IF YES: what made you feel respected?

COMMUNICATION WITH YOUR PROVIDER IS ONE OF THE MOST IMPORTANT ASPECTS OF HEALTHCARE.

What is your preferred way to communicate about your health? (e.g., verbally, interpreter, patient portal, through a caregiver)

How did the doctor/physician assistant/nurse practitioner communicate with you? Did you tell them you preferred to communicate in this way?

IF NOT, what is your preferred communication method?

IF YES, what did you like about it?

COMFORT & KNOWLEDGE AROUND HEALTH CONDITION

Did you feel that the doctor is knowledgeable and comfortable about your health conditions and health needs?

IF NOT, what did you feel was needed?

IF YES, what examples can you provide that demonstrated knowledge and comfort?

SOCIAL NEEDS & COMMUNITY RESOURCES

Many patients at Michigan Medicine experience difficulties with getting quality food, housing, transportation, or being socially isolated. Have you or someone in your community experienced these types of difficulties?

PROBE: How do you/they try to overcome these difficulties?

PROBE: Have you/they reached out to community resources for help?

How do you think these difficulties impact people’s health?

How do you think Michigan Medicine, and other hospitals and healthcare organizations, can help with these difficulties? (Note: Regardless of experiencing them or not.)

Do you have any other thoughts about these types of difficulties?

Is there anything else you would like to share with us about the care you receive at [clinic name] Family Medicine?
