# Supplementary material for: Model Clinic to Increase Preventive Screenings Among Patients With Physical Disabilities: Protocol for a Mixed Methods Intervention Pilot Study
Source: JMIR Res Protoc. 2023 Oct 25;12:e50105. doi: 10.2196/50105 (PMC10632921; doi:10.2196/50105)
Supplement: Multimedia Appendix 4 [file resprot_v12i1e50105_app4.docx]

**Appendix 4: Provider Interview Questions**

**Primary prompt:** What is your experience with using the PWPD BPA?

**Secondary prompts:**

| CFIR construct | Interview Question |
| --- | --- |
| Characteristics of individuals: Knowledge and beliefs about intervention | 1. Can you describe the Patients with Physical Disabilities BestPractice Advisory? |
| Characteristics of individuals: Knowledge and beliefs about intervention | 2. How do you feel about the Patients with Physical Disabilities BestPractice Advisory being used in Briarwood Family Medicine? |
| Implementation climate: Tension for change | 3. Do you think that the PWPD BPA is needed? |
| Implementation climate: Relative priority | 4. With multiple aspects of care being delivered, what level of priority do you think Briarwood Family Medicine gives to implementing the PWPD BPA? |
| Implementation climate: Compatibility | 5. How well does the PWPD BPA fit in existing workflows? |
| Characteristics of individuals: Self-efficacy | 6. How confident do you think your colleagues feel about using the PWPD BPA? |
| Characteristics of individuals: Individual stage of change | 7. How prepared are you to use the PWPD BPA? |
| Multiple constructs (e.g., Intervention characteristics – Design quality & packaging; Intervention characteristics – Complexity) | 8. What are the barriers to using the PWPD BPA when working with a patient?  (e.g., complexity, knowledge and information, time) |
| Process: Engaging – Intervention participants | 9. How do you communicate with patients about the recommendations on the PWPD BPA? |
| Implementation climate: Goals & feedback, | 10. Do staff at Briarwood Family Medicine communicate about the PWPD BPA (e.g., setting goals, getting feedback from colleagues)? |
