# Supplementary material for: Model Clinic to Increase Preventive Screenings Among Patients With Physical Disabilities: Protocol for a Mixed Methods Intervention Pilot Study
Source: JMIR Res Protoc. 2023 Oct 25;12:e50105. doi: 10.2196/50105 (PMC10632921; doi:10.2196/50105)
Supplement: Multimedia Appendix 5 [file resprot_v12i1e50105_app5.docx]

**Table S1.** Outcomes and models for the intervention effectiveness analysis.

| Outcome | Variable type | Suggested model | Predictors |
| --- | --- | --- | --- |
| Preventative screening biomarkers, eg, HbA_1c_ | Continuous | Linear model | - Binary intervention variable - Binary time variable - Interaction between intervention and time, ie, DD estimator - Time-invariant variables (eg, sociodemographic variables listed in Textbox 2) |
| PHQ-9 (overall score) |  |  |  |
| GAD-7 (overall score) |  |  |  |
